# The Association between Genes Polymorphisms of Heparan Sulfate Proteoglycan 2 (*HSPG2*) and Chondroitin Sulfate Proteoglycan 2 (*CSPG2*) and Intracranial Aneurysm Susceptibility: A Meta-Analysis

**Published:** 2019-11

**Authors:** Huahui LIU, Wen HUANG

**Affiliations:** Department of Neurology, First Affiliated Hospital of Guangxi Medical University, Nanning, 530021, China

**Keywords:** SNP, *HSPG2*, *CSPG2*, Intracranial aneurysm, Meta-analysis

## Abstract

**Background:**

We aimed to investigate whether the polymorphisms of gene heparan sulfate proteoglycan 2 (*HSPG2*) and chondroitin sulfate proteoglycan 2 (*CSPG2*) are associated with increased risk of intracranial aneurysms (IAs) susceptibility.

**Methods:**

The Cochrane Library, Medline, PubMed, and Embase databases were carefully searched for potential researches before Mar 30, 2018. The title, abstract, and full text were assessed to determine whether the paper was suitable for inclusion. The pooled odds ratios (ORs) with 95% confidence intervals (CIs) were presented to assess the associations between *CSPG2* and *HSPG2* polymorphisms and IAs susceptibility.

**Results:**

We enrolled 7 papers, 4 matched single nucleotide polymorphisms (SNPs) of *CSPG2* (rs173686, rs251124) or *HSPG2* (rs173686, rs251124), and a total of 8651 participations (3674 with IAs and 4977 for control). For the rs251124 polymorphism of *CSPG2*, the quantitative synthesis from 5 studies showed significant difference in the gene allele comparison of T vs. C (OR, 1.129; 95% CI, 1.029, 1.238; *P*=0.01). Similar results were found for rs3767137 of *HSPG2* (A vs. G, OR, 0.842, 95% CI*=*0.759–0.935, *P=*0.001). However, for the rs173686 polymorphism of *CSPG2* and rs7556412 polymorphism of *HSPG2*, no significant difference was found (*P=*0.259 and *P=*0.474, respectively)

**Conclusion:**

The SNPs rs251124 of *CSPG2* and rs3767137 of *HSPG2* had statistically significant associations with IAs susceptibility. The minor allele T of rs251124 demonstrated a harmful effect but the minor allele A of rs3767137 demonstrated a protective role with regard to the risk of IAs. However, no such associations were found in the SNPs rs173686 of *CSPG2* and rs7556412 of *HSPG2*.

## Introduction

Intracranial aneurysms (IAs) affect approximately 2% of the general population and account for 85% of subarachnoid hemorrhage (SAH) (1, 2). IA rupture is common at the age of 40–60 yr and the prognosis after rupture is poor: about fifty percent of the cases die and another twenty percent cases remain disability after treatment and rehabilitation (3–5).

Various theories have been proposed for the development of IA, and IA is always considered as a multifactorial disorder due to the interaction of both environmental and genetic factors. Environmental factors, such as alcohol consumption, smoking, and hypertension seem to be common IA risk factors (6, 7). However, the genetic factors cannot be excluded for the risk of IAs. A familial predisposition was the strongest risk factor for the development of IA, with the risk of rupture increased to 7-fold (8). Another research also reported a four-fold risk of aneurismal rupture in a first degree relative among the familial clustering of IAs (9). Similar conclusion was detected in studies of twins. Moreover, the gene factors that result in the changes of vascular extracellular matrix (ECM) may also be an important reason for IAs susceptibility. ECM remodeling plays an important role in maintaining the structure and integrity of the arterial wall. Reduced extracellular matrix is a prominent feature of cerebral aneurysms.

Previous papers have explored a list of genes involved in the maintenance of the integrity of the ECM with case-control studies (10, 11). Among which, the heparan sulfate proteoglycan 2 (*HSPG2*) and chondroitin sulfate proteoglycan 2 (*CSPG2*) genes have been identified as susceptibility genes for IA (12, 13). However, the association between the SNPs of *CSPG2* and *HSPG2* and risk of IAs remains argumentative due to the contrary of the reported researches. The importance of SNPs of *CSPG2* and *HSPG2* in the etiology of IA would be strengthened if the associations could be confirmed in large and various populations. The present study aimed to detect the relationships between various SNPs of *CSPG2* and *HSPG2* and risk of IAs susceptibility via meta-analysis and system review.

## Methods

The Cochrane Library, Medline, PubMed, and Embase databases were carefully searched independently by L.H.H and H.W to detect relevant studies published before Mar 30, 2018. The search criteria “VCAN or chondroitin sulfate proteoglycan 2 or *CSPG2* or proteoglycans perlecan or heparan sulfate proteoglycan 2 or *HSPG2*” and “intracranial aneurysm” were used for text word searches. The “related articles” function was used for potentially additional articles. The reference lists of the selected articles were also manually examined to find relevant studies that were not discovered during the above-mentioned database searches. The language was restricted to only English.

All published case-control studies reporting genetic polymorphisms of “*CSPG2* or *HSPG2*” in IAs was potentially enrolled in the present research. All the papers were assessed carefully for eligibility with the titles, abstracts and finally full papers. When several papers from the same study were published, only the most recent or informative one was included.

Data extraction:

The data extraction of all outcomes and variables of interest were performed independently by L.H.H and H.W. Disagreements were resolved through discussion and consensus. Data on author affiliation, number of participants, patients’ data and genotyping information were extracted. If insufficient data were reported, we contacted corresponding authors for additional information and the paper was excluded if there was no response.

### Quality assessment

The quality assessment of the included studies was performed independently by the two reviewers L.H.H and H.W using the Newcastle-Ottawa Scale (NOS). The NOS employed a star rating system to assess quality from 3 broad perspectives of the study: 1) selection of the study groups, 2) comparability of the groups, and 3) identification of the exposure (for case-control studies) or outcome of interest (for cohort studies). Scores ranged from 0 to 9 stars, and studies with no less than 7 stars were considered to be of high quality.

### Statistical analysis

The statistical analysis was performed with the software named ‘‘Comprehensive Meta-Analysis (Version 2.2, Englewood, USA)’’. The association strength between the gene polymorphisms and IAs susceptibility was calculated by the Z test, presented with the OR (respective 95% CIs). And the significance of the pooled OR was determined with *P*-values (less than 0.05 was considered significant). I^2^ statistics, with the value ranged from 0% to 100% (complete consistency to complete inconsistency), was used to determine the statistical heterogeneity among studies. If the I^2^ -value was more than 50%, the random-effects model was chosen to calculate the pooled OR; otherwise the fixed-effects model was used. All of the results were presented as forest plots.

## Results

**Fig. 1:**
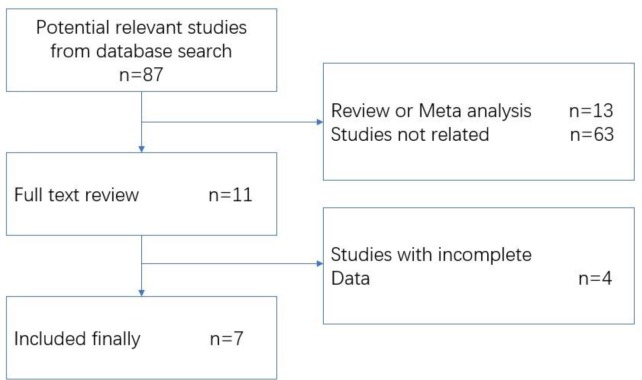
Search strategy flow diagram

The initial literature search retrieved 87 relevant articles (duplicates were discarded), and finally, 7 studies were included in the present study after carefully review. Flowcharts describing the study selection was shown in Fig. 1.

Overall, 8651 participations (3674 with IAs and 4977 for control) were enrolled in the studs. All the patients were radiologically confirmed intracranial aneurismal cases and the controls were age and gender matched. Table 1 listed the basic information of the included studies. Only 4 SNPs of the two genes (rs173686, rs251124, rs3767137, and rs7556412) were matched across at least three studies and were finally included in the study. The review of the information and data extraction revealed 100% agreement between the 2 reviewers.

**Table 1:** Characteristics of the individual studies included in the meta-analysis

***Pmid***	***Country***	***Year***	***Author***	***Inclusion***		***Number***		***Matched snps***
						***Case***		***Control***
25606449	India	2014	Moinak banerjee	Radiologically confirmed intracranial aneurismal cases		220	250	Rs251124, rs173686
23568740	China	2013	Lijuan hu	Radiologically confirmed intracranial aneurismal cases		537	1071	Rs251124, rs3767137
20053631	Netherlands	2010	Ynte m. Ruigrok	Radiologically confirmed intracranial aneurismal cases	Stage 1	376	648	Rs173686, rs3767137, rs7556412
					Stage 2	360	376	
19506372	Japan	2009	Boris krischek	Radiologically confirmed intracranial aneurismal cases		632	808	Rs251124, rs3767137
18484070	China	2007	Jizong zhao	Radiologically confirmed intracranial aneurismal cases		240	240	Rs251124, rs173686
17038484	Netherlands	2006	Ynte m. Ruigrok	Radiologically confirmed intracranial aneurismal cases	Stage 1	382	609	Rs3767137, rs7556412
					Stage 2	310	336	
16917090	Netherlands	2006	Ynte m. Ruigrok	Radiologically confirmed intracranial aneurismal cases	Stage 1	307	639	Rs251124, rs173686
					Stage 2	310		

### Main analysis

The genotype and allele frequencies of the *CSPG2* and *HSPG2* gene variants between patients and controls were extracted and analyzed from the included studies. Only the meta-analysis of the allele frequencies was able to conduct between the studies, the meta-analysis of the genotype frequencies was not available because of the limited data. The meta-analysis for the relationship between gene *CSPG2* (rs173686, rs251124) or *HSPG2* (rs173686, rs251124) polymorphisms and IAs susceptibility were shown in Table 2.

**Table 2:** Meta-analysis of associations between *CSPG2* and *HSPG2* polymorphisms and IAs susceptibility

***Gene***	***SNP***	***Number***		***Effect model***	***A vs. A***	
		***Case***	***Control***		***OR (95% CI)***	**P*-values***
*CSPG2*	Rs173686	1442	1778	Random	1.110 (0.926– 1.332)	0.259
*CSPG2*	Rs251124	2227	2845	Random	1.129 (1.029– 1.238)	0.01
*HSPG2*	Rs3767137	1700	2401	Fixed	0.842 (0.759– 0.935)	0.001
*HSPG2*	Rs7556412	1068	1593	Random	1.063 (0.899– 1.258)	0.474

**Fig. 2:**
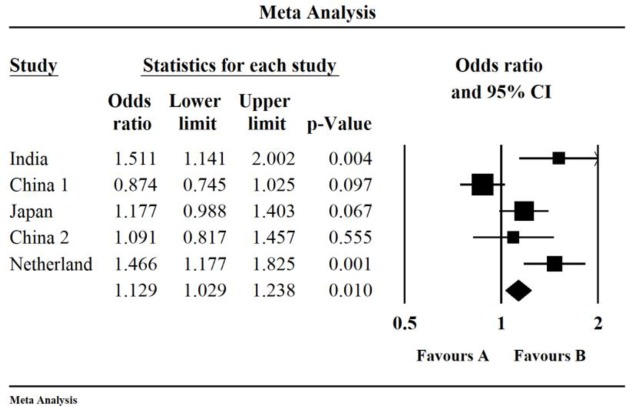
The forest plots present the association between polymorphism of rs251124 and IAs susceptibility. Number of included studies: n*=*5; OR, 1.129; 95% CI, 1.029, 1.238; *P=*0.01)

For the rs251124 polymorphism of *CSPG2*, the quantitative synthesis from 5 studies showed the gene allele significant difference in the gene allele comparison of T vs. C (OR, 1.129; 95% CI, 1.029, 1.238; *P*=0.01) (Fig. 2). However, for the rs173686 polymorphism of *CSPG2*, no significant difference was found in the gene allele comparison (*P=*0.259).

**Fig. 3:**
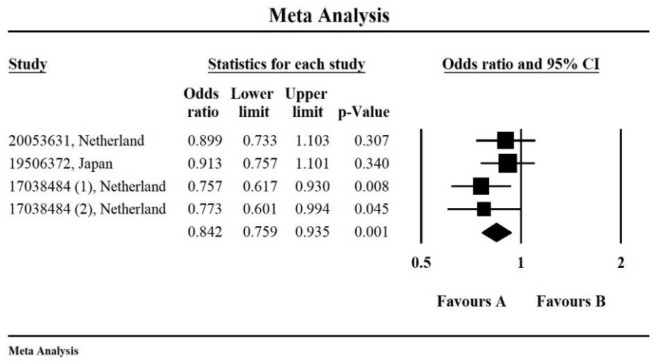
The forest plots present the association between polymorphism of rs3767137 and IAs susceptibility. Number of included studies: n=4; OR, 0.842, 95% CI*=*0.759–0.935, *P=*0.001)

Similarly, for SNPs rs3767137 of *HSPG2*, quantitative synthesis showed significant differences in the comparisons of allele frequencies (A vs. G, OR, 0.842, 95% CI*=*0.759–0.935, *P=*0.001, Fig. 3). And no significant difference was found in the gene allele comparison for the rs7556412 polymorphism of *HSPG2* (*P=* 0.474).

### Publication bias

Egger's test was performed to access the publication bias of studies included in this meta-analysis. The Egger's test demonstrated no evidence of publication bias for all the four SNPs.

## Discussion

The study demonstrated the SNPs rs251124 of *CSPG2* and rs3767137 of *HSPG2* had statistically significant associations with IAs susceptibility. The minor allele T of rs251124 demonstrated a harmful effect but the minor allele A of rs3767137 demonstrated a protective role with regard to the risk of IAs. However, no such associations were found in the SNPs rs173686 of *CSPG2* and rs7556412 of *HSPG2*. Because of the increasing number of cases and controls in this analysis, the estimates of association obtained are more precise.

The pathology of IA is the ballooning of intracranial arteries that is easy to rupture or constrict the nerves. IAs is caused by weakening of arterial walls, which indicates the role of defective ECM in IA pathogenesis. Gene *CSPG2* and *HSPG2* encode two important proteins in maintains of the ECM, thus the genotype variations of *CSPG2* and *HSPG2* and the resulting functional changes may be an important part of IAs pathologies. Until now, contrary results were reported with regard to the relationship between *CSPG2* and *HSPG2* polymorphisms and IAs susceptibility. In the Dutch population, SNPs in strong linkage disequilibrium and haplotypes constituting these SNPs in the *CSPG2* gene were associated with IAs (12), which suggested that variation in or near the *CSPG2* gene played a role in susceptibility to IAs. In another study, the polymorphisms of *HSPG2* gene were also found to be involved in the increased risk of IAs (10). Similarly, another study replicated the association of *CSPG2* and risk of IAs, and there is a trend for *HSPG2* toward association by meta-analysis (14). On the contrary, replication studies reported a lack of association of the *CSPG2* and *HSPG2* variants with IA susceptibility (15, 16). In addition, the different SNPs of the *CSPG2* and *HSPG2* genes also demonstrated different results, showing not all SNPs could influent the function of the proteins.

Gene *CSPG2*, (17), also known as versican (VCAN), localized on 5q12–q14, is a member of the aggrecan/versican proteoglycan family. The protein encoded is a large chondroitin sulfate proteoglycan and is a major component of the ECM. This protein is involved in cell adhesion, proliferation, migration, and angiogenesis and also plays a vital role in tissue maintenance and morphogenesis (18). *HSPG2* gene, located on chromosome 1p34.3-p36.13, encodes the perlecan protein that consists of a core protein and three long chains of glycosaminoglycans (19). The perlecan protein is a large multidomain proteoglycan that binds to and cross-links many ECM components and cell-surface molecules. Perlecan is a key component of the vascular extracellular matrix, where it helps to maintain the endothelial barrier function. It can also promote growth factor (e.g., FGF2) activity and thus stimulate endothelial growth and re-generation. Both proteins encoded by the two genes are central parts of the ECM and thereby contribute to the maintains of the vascular wall (18).

Though the present study explored the relationship between *CSPG2* and *HSPG2* polymorphisms and IAs susceptibility successfully. The present study still had some limitations that could not be ignored. First, the inconsistency of the baseline characteristics and the publication bias between the case and control groups may have distorted the meta-analysis. Second, only 4 SNPs were able to be included in the final meta-analysis, remaining much more of previous reported SNPs controversy, with regard to a large number of variants found for these two genes. In addition, the possibility of synergistic action to IAs susceptibility by haplotypes could not be ignored, however, the present meta-analysis is not able to explore it.

## Conclusion

The SNPs rs251124 of *CSPG2* and rs3767137 of *HSPG2* have had statistically significant associations with an increased risk of IAs susceptibility. The minor allele T of rs251124 showed a harmful effect but the minor allele A of rs3767137 demonstrated a protective role with regard to the risk of IAs. However, no such associations were found in the SNPs rs173686 of *CSPG2* and rs7556412 of *HSPG2*. Because of the increasing number of cases and controls in this analysis, the estimates of association obtained are more precise.

## Ethical considerations

Ethical issues (Including plagiarism, informed consent, misconduct, data fabrication and/or falsification, double publication and/or submission, redundancy, etc.) have been completely observed by the authors.
